# Supplementary material for: Analysing the Relationship between Nutrition and the Microbial Composition of the Oral Biofilm—Insights from the Analysis of Individual Variability
Source: Antibiotics (Basel). 2020 Aug 4;9(8):479. doi: 10.3390/antibiotics9080479 (PMC7460051; doi:10.3390/antibiotics9080479)
Supplement: Supplementary file 1 [file antibiotics-09-00479-s001.zip › additional_file1.pdf]

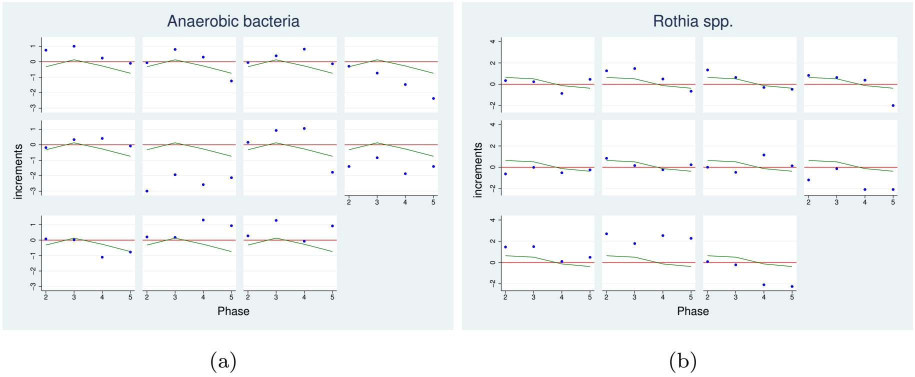

Supplementary Figure. 1 Scatter plot of individual bacterial concentration changes from baseline to each of the other phases. Concentration changes (in log-10 CFU per ml) of anaerobic bacteria (a) and *Rothia* spp. (b) for each of the 11 participants over the phases. Green line: mean value per phase, dot: individual mean value per phase.

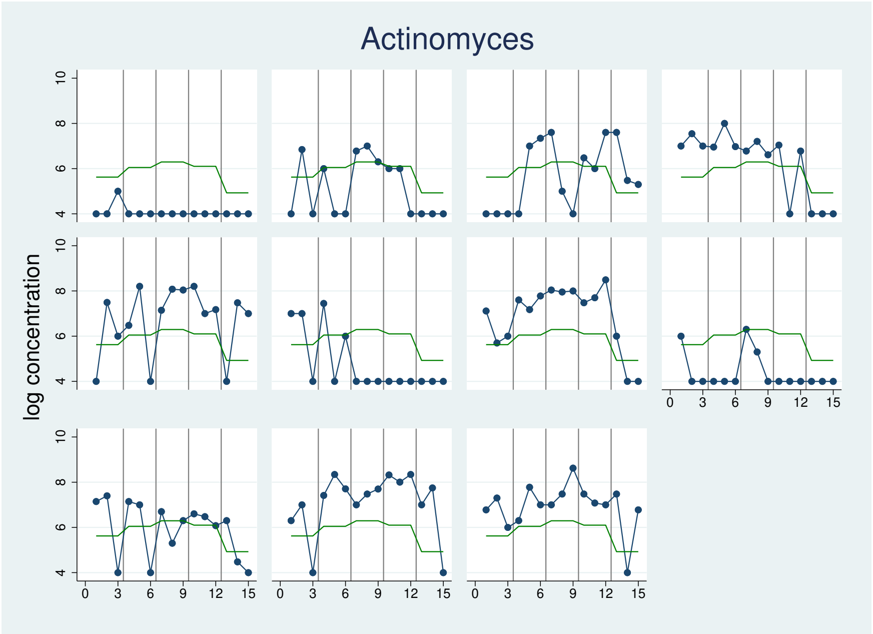

Supplementary Figure. 2 Scatter plot of individual bacterial concentration over time. Concentrations (in log-10 CFU per ml) of *Actinomyces* for each of the 11 participants over the 5 phases. Green line: mean value per phase, grey line: end of the phase.

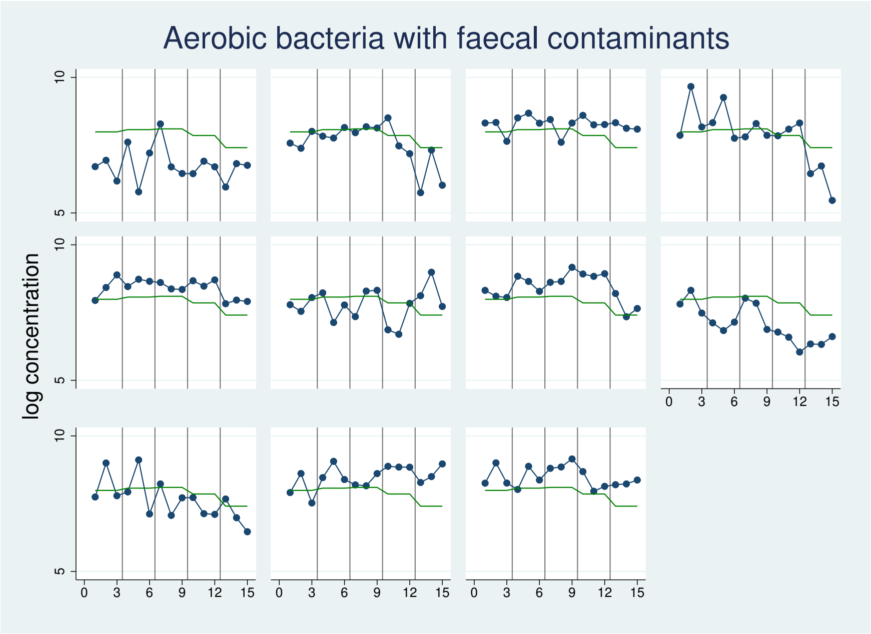

Supplementary Figure. 3 Scatter plot of individual bacterial concentration over time. Concentrations (in log-10 CFU per ml) of Aerobic bacteria wfc for each of the 11 participants over the 5 phases. Green line: mean value per phase, grey line: end of the phase.

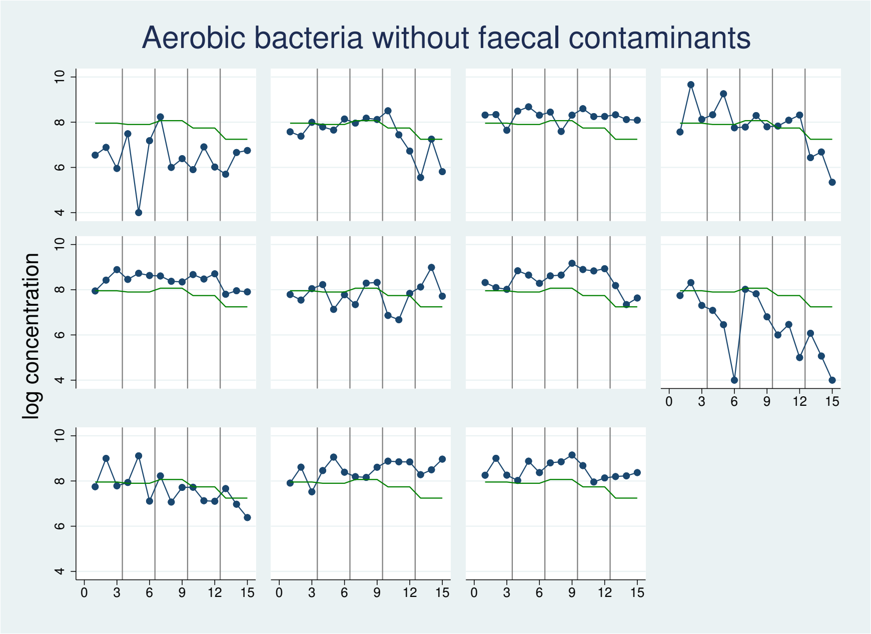

Supplementary Figure. 4 Scatter plot of individual bacterial concentration over time. Concentrations (in log-10 CFU per ml) of Aerobic bacteria fce for each of the 11 participants over the 5 phases. Green line: mean value per phase, grey line: end of the phase.

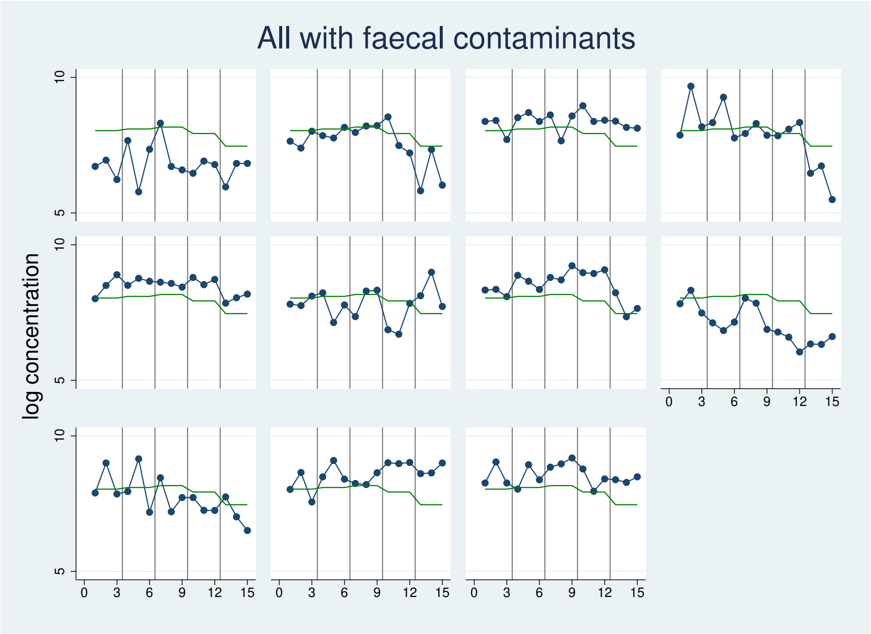

Supplementary Figure. 5 Scatter plot of individual bacterial concentration over time. Concentrations (in log-10 CFU per ml) of All wfc for each of the 11 participants over the 5 phases. Green line: mean value per phase, grey line: end of the phase.

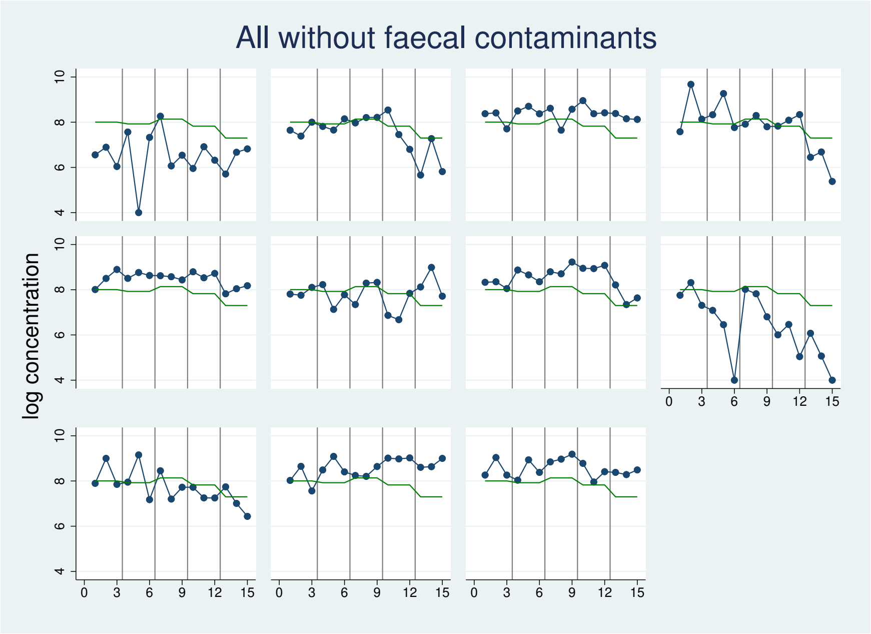

Supplementary Figure. 6 Scatter plot of individual bacterial concentration over time. Concentrations (in log-10 CFU per ml) of All fce for each of the 11 participants over the 5 phases. Green line: mean value per phase, grey line: end of the phase.

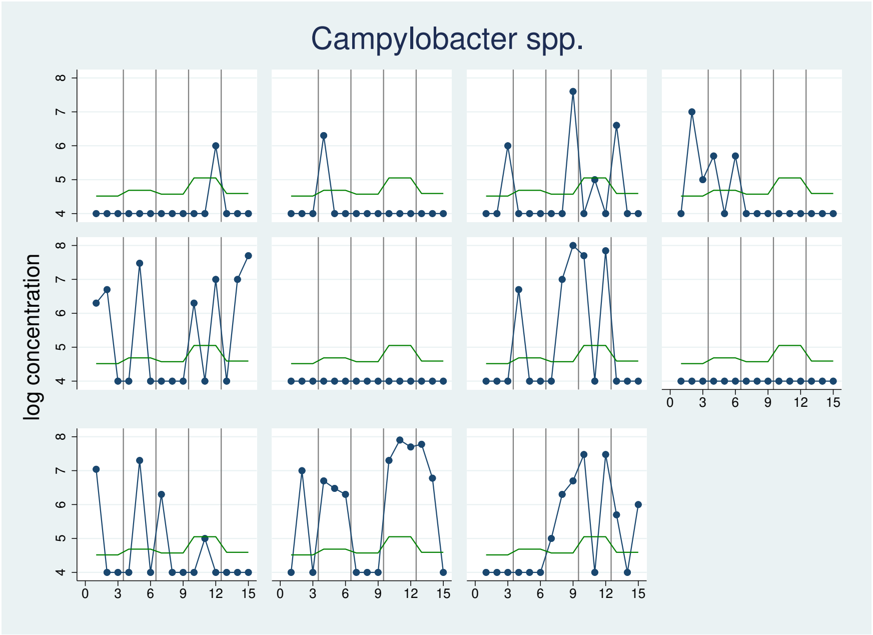

Supplementary Figure. 7 Scatter plot of individual bacterial concentration over time. Concentrations (in log-10 CFU per ml) of *Campylobacter* spp. for each of the 11 participants over the 5 phases. Green line: mean value per phase, grey line: end of the phase.

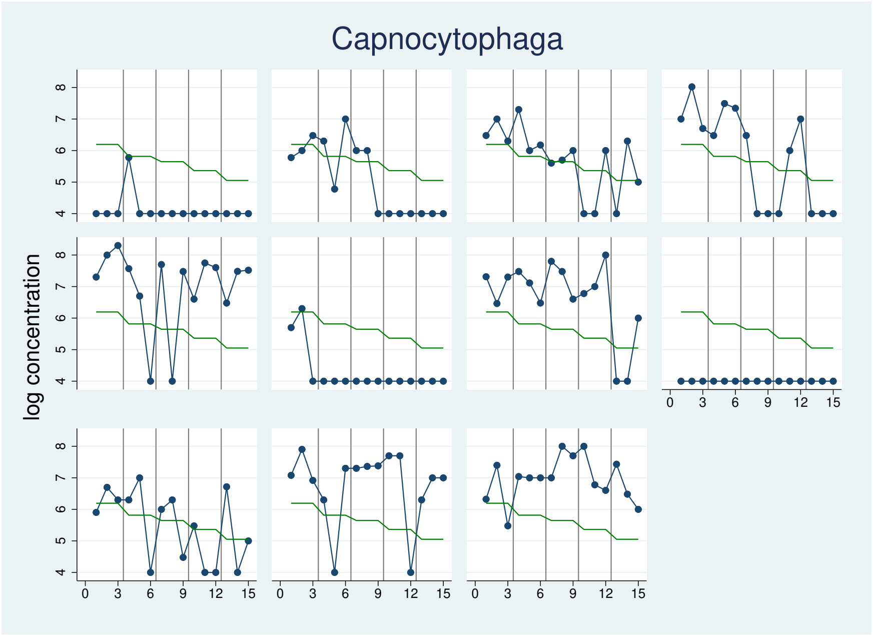

Supplementary Figure. 8 Scatter plot of individual bacterial concentration over time. Concentrations (in log-10 CFU per ml) of *Capnocytophaga* for each of the 11 participants over the 5 phases. Green line: mean value per phase, grey line: end of the phase.

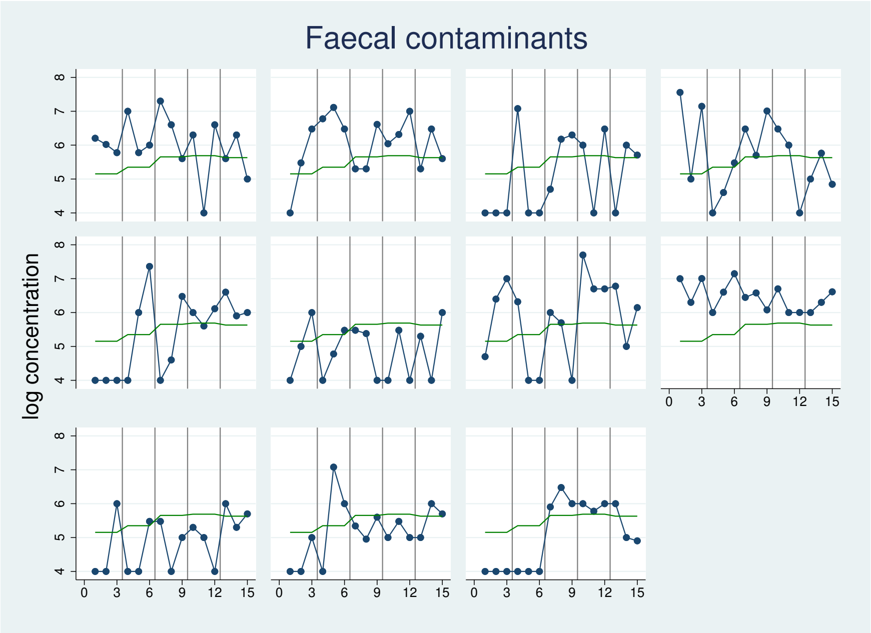

Supplementary Figure. 9 Scatter plot of individual bacterial concentration over time. Concentrations (in log-10 CFU per ml) of Faecal contaminants for each of the 11 participantsover the 5 phases. Green line: mean value per phase, grey line: end of the phase.

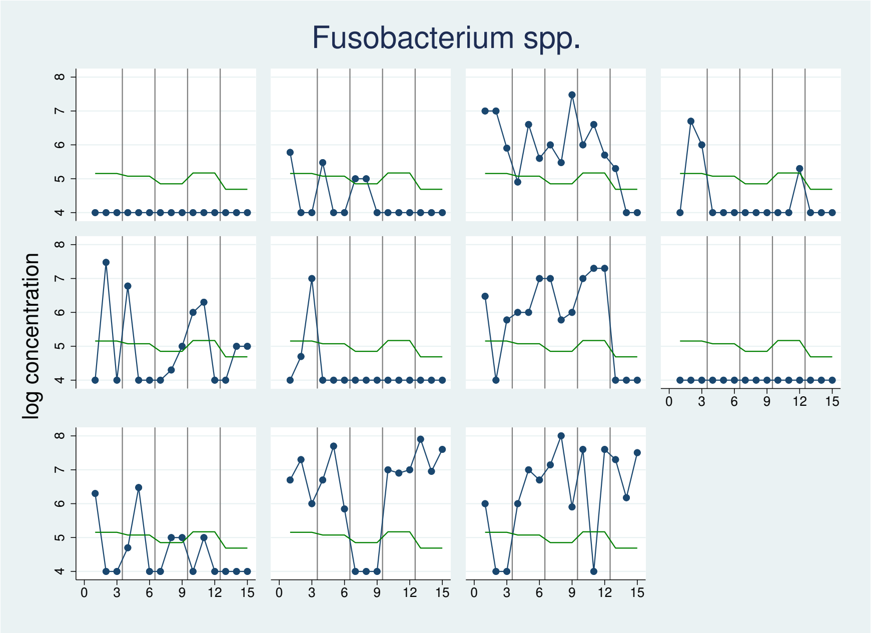

Supplementary Figure. 10 Scatter plot of individual bacterial concentration over time. Concentrations (in log-10 CFU per ml) of *Fusobacterium* spp. for each of the 11 participants over the 5 phases. Green line: mean value per phase, grey line: end of the phase.

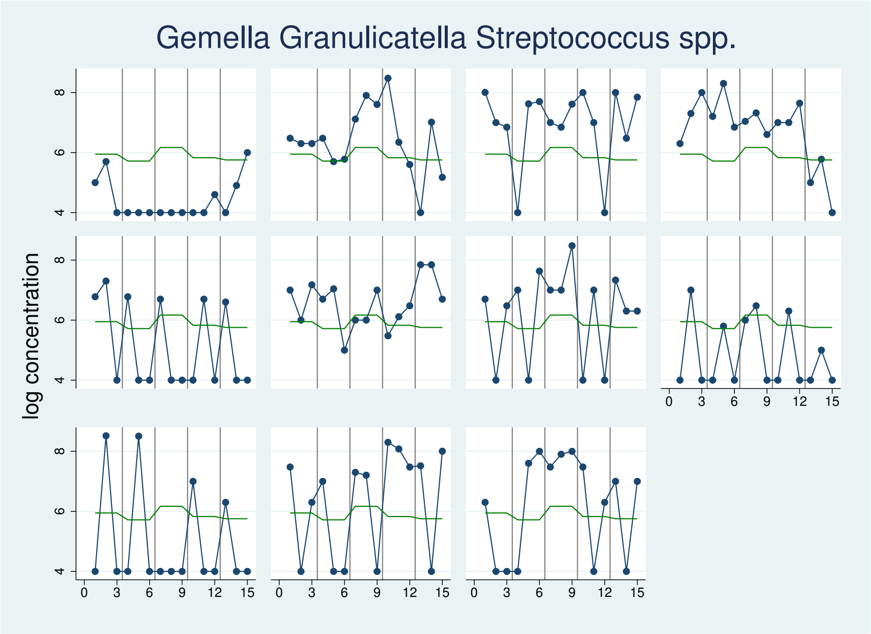

Supplementary Figure. 11 Scatter plot of individual bacterial concentration over time. Concentrations (in log-10 CFU per ml) of *Gemella Granulicatella Streptococcus* spp. for each of the 11 participants over the 5 phases. Green line: mean value per phase, grey line: end of the phase.

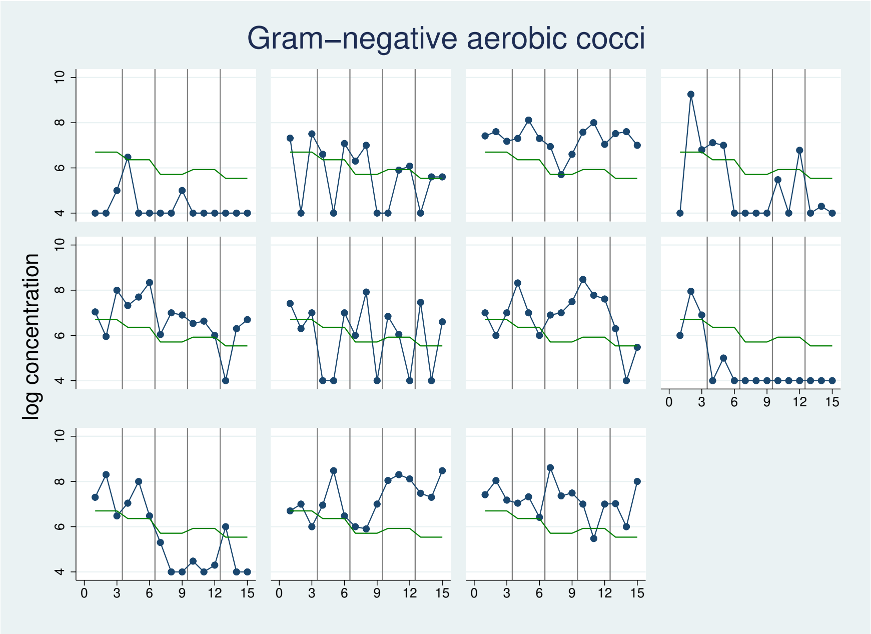

Supplementary Figure. 12 Scatter plot of individual bacterial concentration over time. Concentrations (in log-10 CFU per ml) of Gram-negative aerobic cocci for each of the 11 participants over the 5 phases. Green line: mean value per phase, grey line: end of the phase.

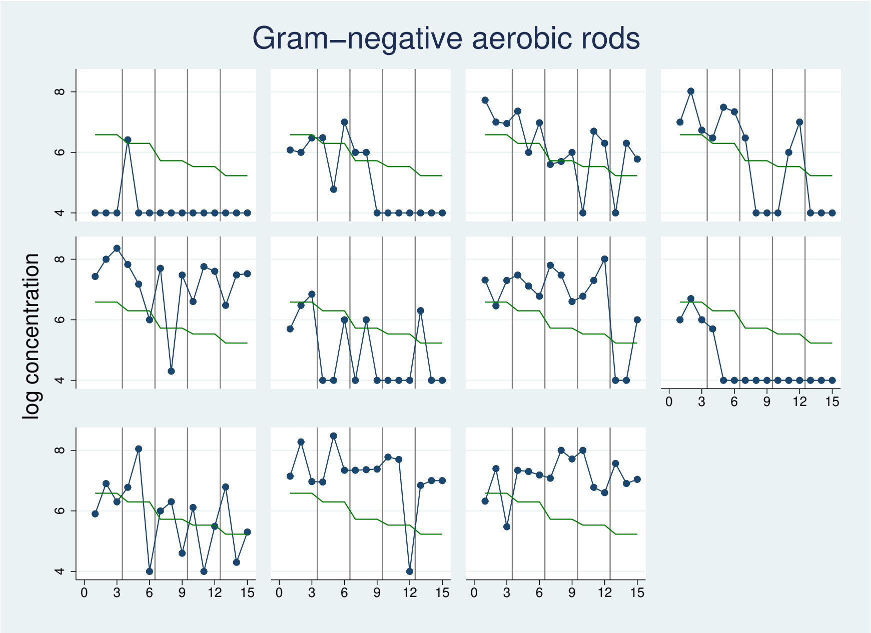

Supplementary Figure. 13 Scatter plot of individual bacterial concentration over time. Concentrations (in log-10 CFU per ml) of Gram-negative aerobic rods for each of the 11 participants over the 5 phases. Green line: mean value per phase, grey line: end of the phase.

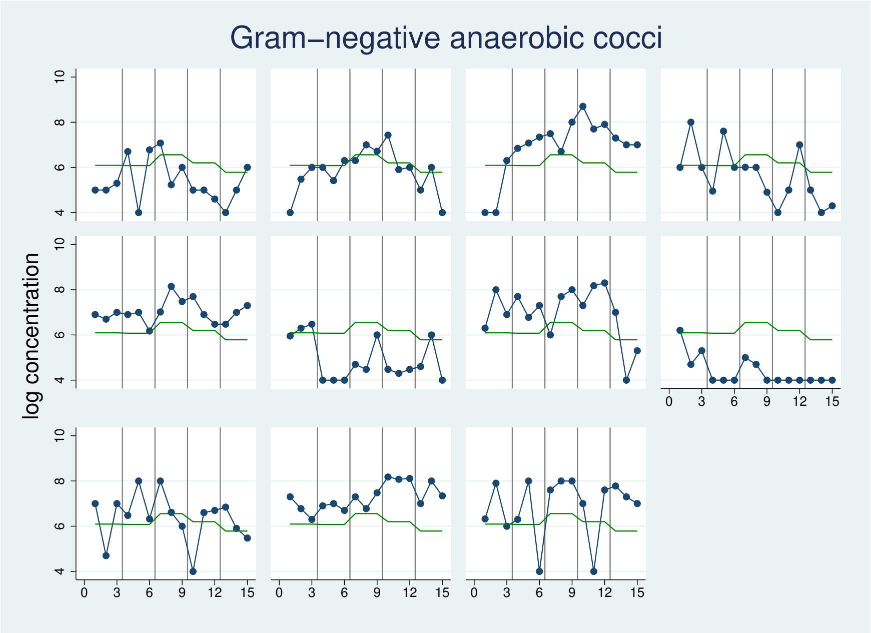

Supplementary Figure. 14 Scatter plot of individual bacterial concentration over time. Concentrations (in log-10 CFU per ml) of Gram-negative anaerobic cocci for each of the 11 participants over the 5 phases. Green line: mean value per phase, grey line: end of the phase.

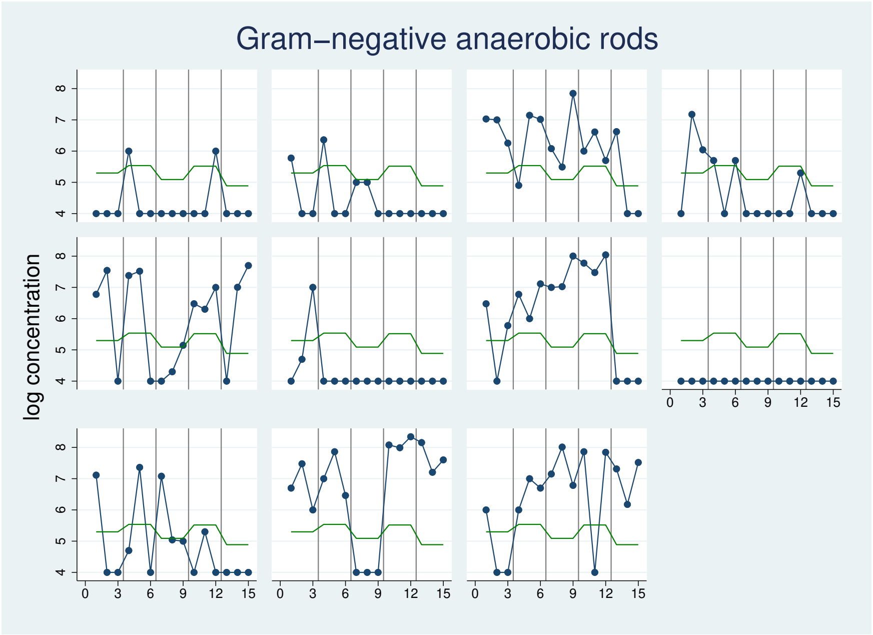

Supplementary Figure. 15 Scatter plot of individual bacterial concentration over time. Concentrations (in log-10 CFU per ml) of Gram-negative anaerobic rods for each of the 11 participants over the 5 phases. Green line: mean value per phase, grey line: end of the phase.

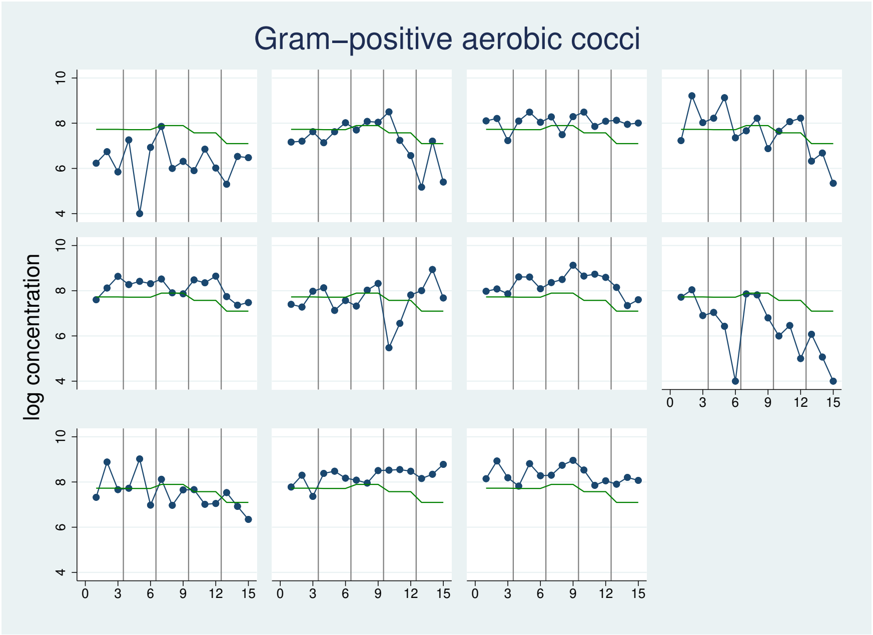

Supplementary Figure. 16 Scatter plot of individual bacterial concentration over time. Concentrations (in log-10 CFU per ml) of Gram-positive aerobic cocci for each of the 11 participants over the 5 phases. Green line: mean value per phase, grey line: end of the phase.

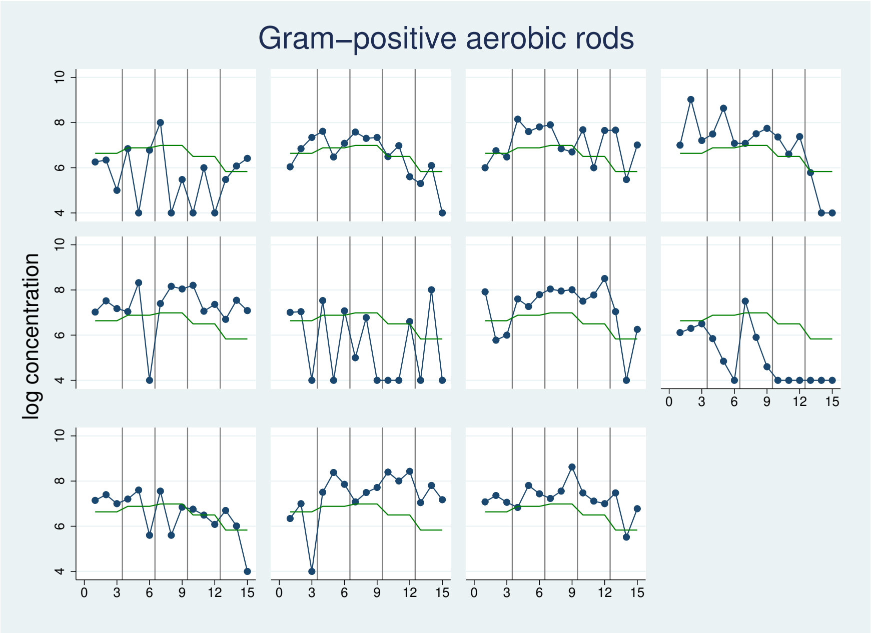

Supplementary Figure. 17 Scatter plot of individual bacterial concentration over time. Concentrations (in log-10 CFU per ml) of Gram-positive aerobic rods for each of the 11 participants over the 5 phases. Green line: mean value per phase, grey line: end of the phase.

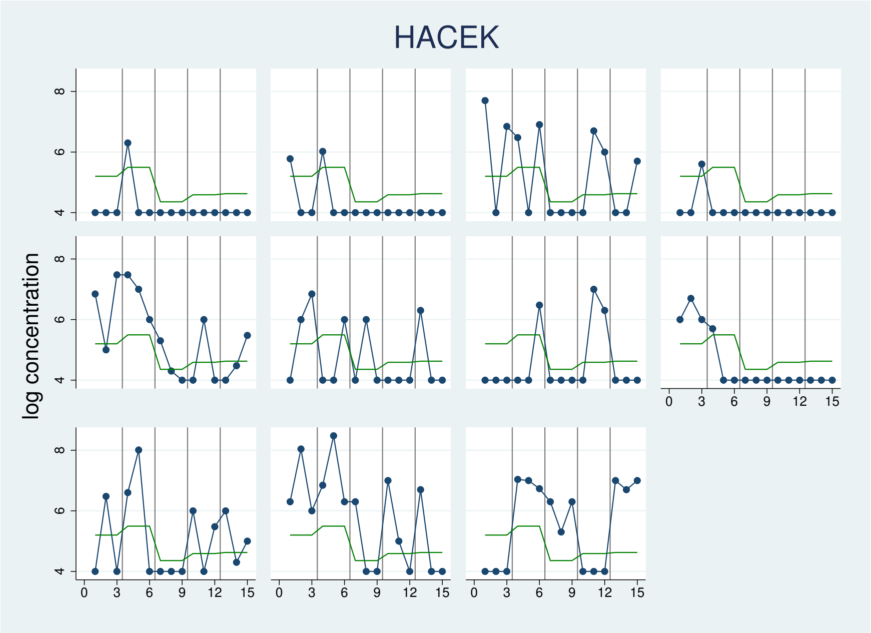

Supplementary Figure. 18 Scatter plot of individual bacterial concentration over time. Concentrations (in log-10 CFU per ml) of Hacek for each of the 11 participants over the 5 phases. Green line: mean value per phase, grey line: end of the phase.

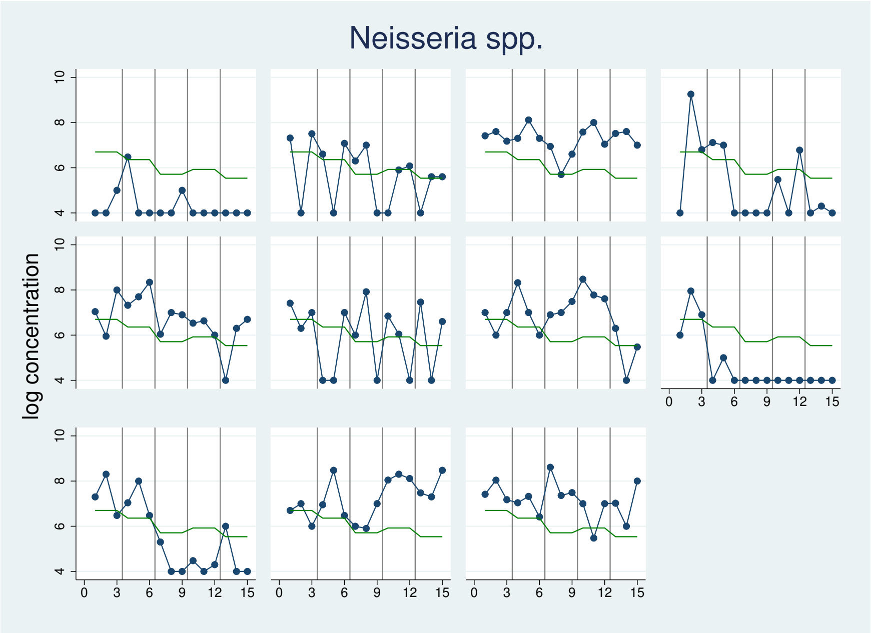

Supplementary Figure. 19 Scatter plot of individual bacterial concentration over time. Concentrations (in log-10 CFU per ml) of *Neisseria* spp. for each of the 11 participants over the 5 phases. Green line: mean value per phase, grey line: end of the phase.

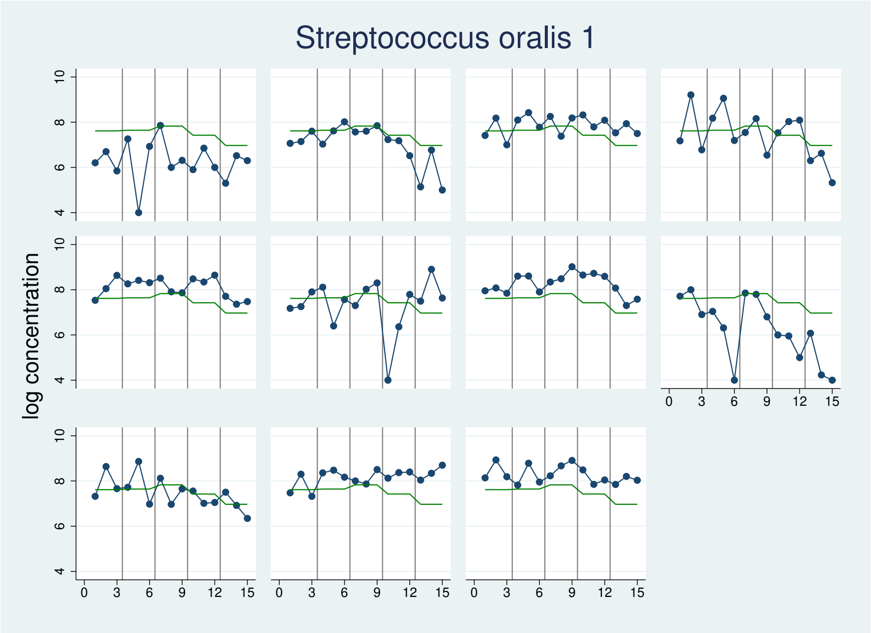

Supplementary Figure. 20 Scatter plot of individual bacterial concentration over time. Concentrations (in log-10 CFU per ml) of *Streptococcus oralis* 1 for each of the 11 participants over the 5 phases. Green line: mean value per phase, grey line: end of the phase.

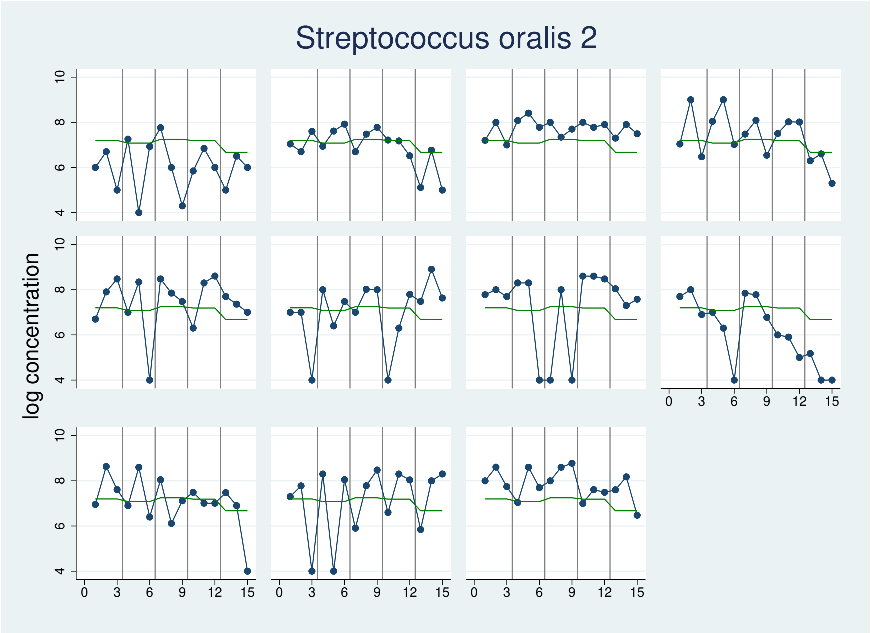

Supplementary Figure. 21 Scatter plot of individual bacterial concentration over time. Concentrations (in log-10 CFU per ml) of *Streptococcus oralis* 2 for each of the 11 participants over the 5 phases. Green line: mean value per phase, grey line: end of the phase.

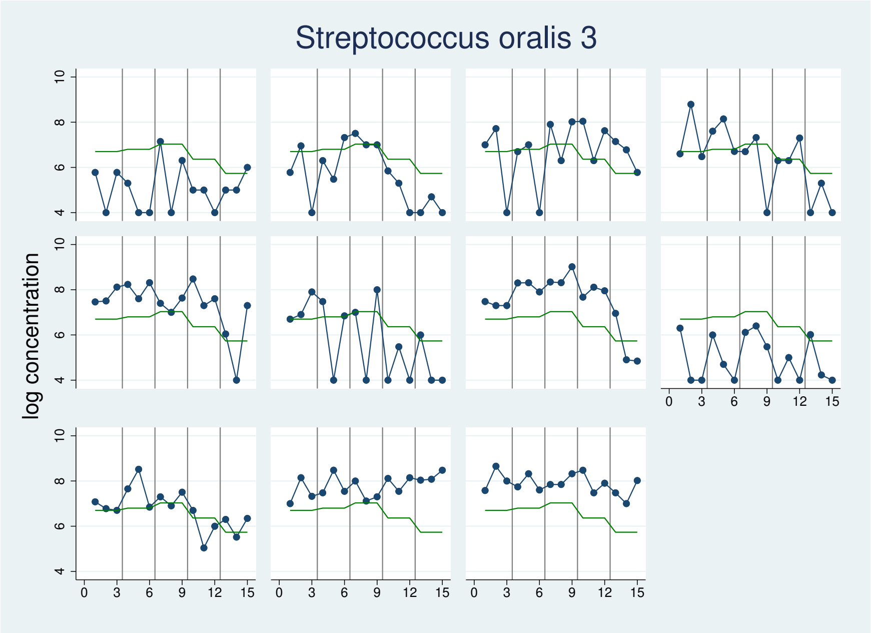

Supplementary Figure. 22 Scatter plot of individual bacterial concentration over time. Concentrations (in log-10 CFU per ml) of *Streptococcus oralis* 3 for each of the 11 participantsover the 5 phases. Green line: mean value per phase, grey line: end of the phase.

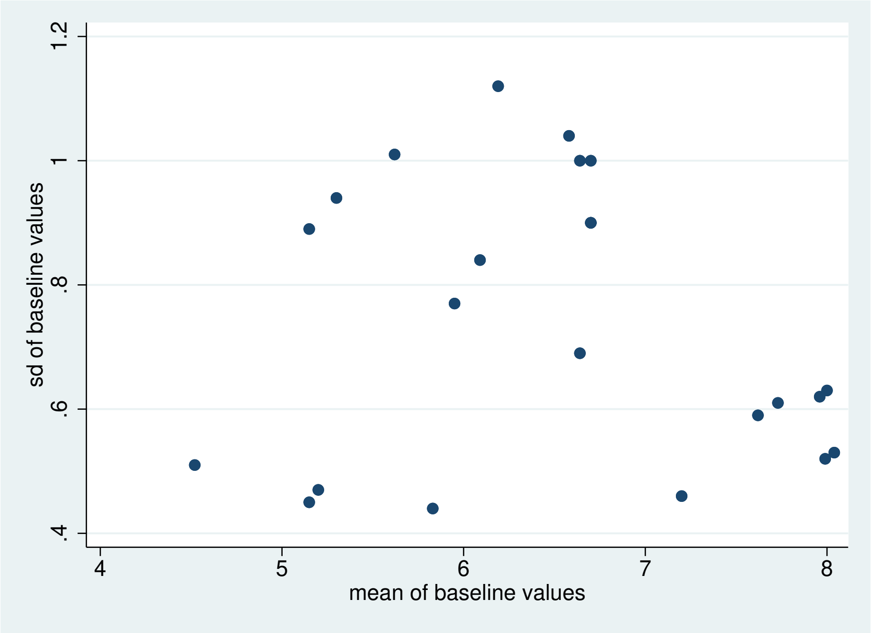

Supplementary Figure. 23 Relation of mean value and standard deviation at initial level.

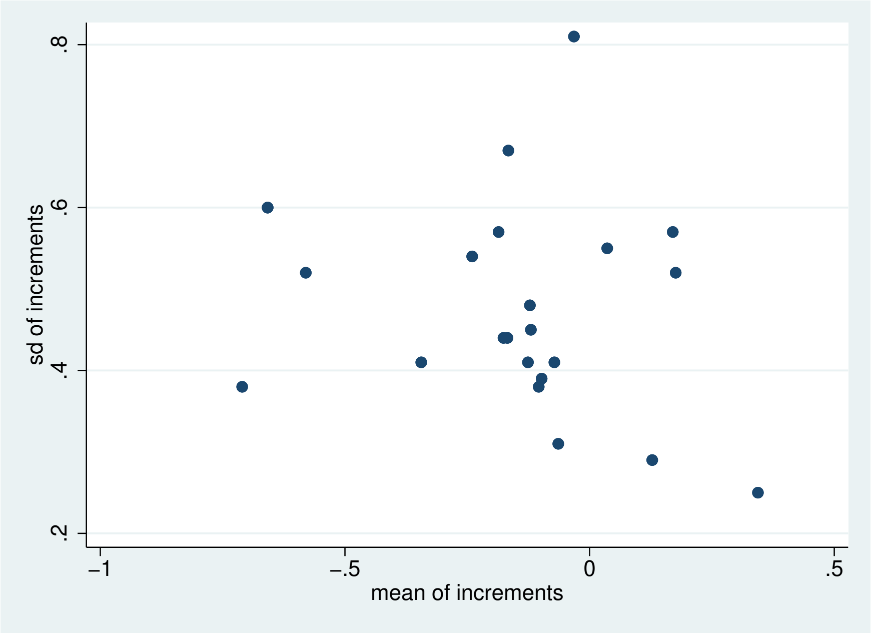

Supplementary Figure. 24 Relation of mean value and standard deviation of increments.
